# Supplementary figures and images for: Impact of coexisting type 2 diabetes mellitus on the urinary microbiota of kidney stone patients
Source: PeerJ. 2024 Feb 26;12:e16920. doi: 10.7717/peerj.16920 (PMC10903351; doi:10.7717/peerj.16920)

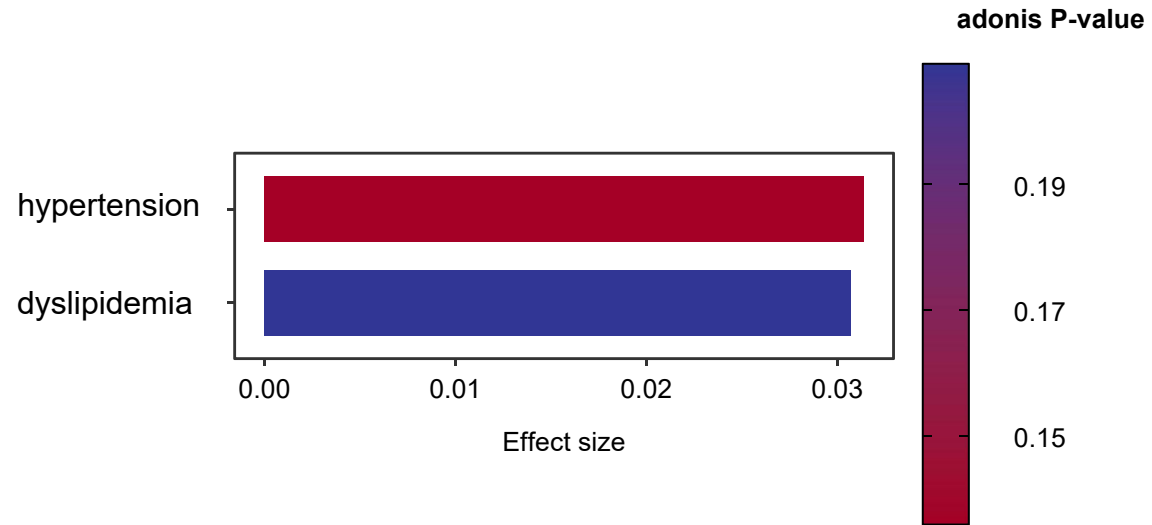

Supplement: Supplemental Information 1 — Principal Coordinate Analysis (PCoA) was conducted based on Bray-Curtis distances at the Amplicon Sequence Variant (ASV) level to explore microbial compositions concerning hypertension and dyslipidemia. Adonis analysis was performed to assess the stability of microbial configurations over varying hypertension and dyslipidemia. [file peerj-12-16920-s001.pdf]
